# How healthy are the healthcare staff in a rural health service? A cross-sectional study

**DOI:** 10.1016/j.ijnsa.2024.100186

**Published:** 2024-02-24

**Authors:** Kristy A Bolton, Penny Fraser, Steven Allender, Rohan Fitzgerald, Susan Brumby

**Affiliations:** a Institute for Physical Activity and Nutrition, Deakin University, Geelong 3220, Australia; b Global Centre for Preventive Health and Nutrition, Institute for Health Transformation, Deakin University, Geelong, Australia; c Western District Health Service, Hamilton, Australia; d School of Medicine, Faculty of Health, Deakin University, Waurn Ponds, Australia; e National Centre for Farmer Health, Western District Health Service, Hamilton, Australia

**Keywords:** Health service, Wellbeing, Healthcare workers, Obesity, Healthy workplace, Rural, Risk factors

## Abstract

**Background:**

The COVID-19 pandemic has amplified the need to understand the health and wellbeing of healthcare workers in hospital settings. Crises like the COVID-19 pandemic create poor health outcomes for healthcare workers, yet little is understood about underlying patterns of modifiable health determinants.

**Objective:**

The aim of the study was to examine the health and wellbeing data of healthcare workers before and during the COVID-19 pandemic and inform future healthy intervention activities within the workplace.

**Design:**

Repeat cross-sectional study pre-2018 and mid-COVID-19 2020.

**Setting(s):**

Rural health service in Victoria, Australia.

**Participants:**

All 800 healthcare workers within the health service were invited; of these, 184 (23%) participated at Time 1 and 87 (11%) at Time 2.

**Methods:**

Diet, physical activity, sleep behaviours, and psychological distress were collected via online survey in Qualtrics. Pre-COVID-19 pandemic, anthropometry (height, weight, waist circumference, blood pressure), and type 2 diabetes risk were collected by a trained practitioner. However, to reduce burden on healthcare workers mid-COVID-19 pandemic, only self-reported anthropometry was collected.

**Results:**

The majority of participants were Australian-born females (84% at both timepoints) with half over the age of 45 (63% Time 1, 53% Time 2). Around half worked part-time (49% Time 1, 54% Time 2), over a third full-time (39% Time 1, 36% Time 2), and the majority reported working regular day shifts in the past 3 months (70% Time 1, 65% Time 2). Among this sample, there were few smokers (9% Time 1, 7% Time 2), and two thirds of participants were living with overweight or obesity (64% Time 1, 67% Time 2). Across both time points, compliance with health guidelines was low; 41% (Time 1) and 42% (Time 2) met fruit, 17% (Time 1) and 12% (Time 2) met vegetable, and just under 50% met physical activity guidelines at both time points. Those reporting moderate to very high levels of psychological stress increased from 42% (Time 1) to 59% (Time 2) (*p<* 0.05). At Time 1, >80% were at intermediate (39%) or high (33%) risk of developing type 2 diabetes within the next 5 years; and a third (32%) were hypertensive. Reasons for physical inactivity at work included already exercising out-of-work hours (28%), living too far from work (18%), available time (9%), and inflexible work hours (8%).

**Conclusions:**

Several high-risk health areas along with opportunities for supporting improved wellbeing were identified in this group of healthcare workers. Psychological distress of healthcare workers urgently needs to be addressed, and this is reinforced by the need to understand the longitudinal impact of COVID-19 on the health and wellbeing of healthcare workers. Workplace settings, such as a health service, is an ideal setting to invest in healthcare workers with individual, organisational, and broader community benefits.

Registration: Nil (not a clinical trial)

Tweetable abstract: The health of healthcare workers needs to be prioritised for individual, organisational, and broader community benefits.

What is already known about this topic

• Healthcare workers operate in a stressful environment; with rotating shifts impacting health and recent evidence suggesting health service/hospital staff have sub-optimal dietary and physical activity behaviours.
• Workplace settings are an ideal place to promote healthy behaviours amongst staff.
• The COVID-19 pandemic has amplified the need to understand the health and wellbeing of healthcare workers in hospital settings.

What this paper adds

• Similar to the general population, in this population of rural healthcare workers, there was low compliance with dietary and physical activity guidelines.
• Despite a smaller sample size at Time 2, there was a significant increase in psychological distress reported.
• This data can be used to inform the development of targeted intervention activities, specifically for this cohort of healthcare staff and in this workplace setting.

## 1 Background

Workplaces are a priority setting to promote healthy behaviours for many reasons (World Health Organization, 2020). Workplace infrastructure can support promotion of health due to the fact that there is wide reach and the acknowledgement that workplaces influence physical, mental, economic, and social wellbeing of staff, their families, communities, and society (World Health Organization, 2020). Healthcare has one of the largest working populations in Victoria and Australia (Australian Bureau of Statistics, 2019). There is an expectation that nursing staff, in particular, have healthier lifestyles compared to the general population, as they are engaged in health promotion and caring for those who are ill (Schneider et al., 2019).

However, this is not always the case. Nurses, for example, may not be practicing or able to practice what they preach with regards to healthy lifestyles (Blake et al., 2011). Shift work, in particular, has been associated with risk of overweight and obesity, poor nutrition (e.g., unhealthy snacking), poor physical activity, and disturbed sleep (James et al., 2017), making nursing and health professionals a vulnerable population for the development of adverse health outcomes. Health professionals in hospital settings have demanding roles, which result in poor health outcomes, such as musculoskeletal back pain due to physical requirements on the job (Genc et al., 2016); high stress due to shift work (Aneni et al., 2014; Belkic and Nedic, 2012); fatigue, sleep deprivation, low job satisfaction, and burnout; all of which can contribute to high staff turnover (Alameddine et al., 2017; Blake et al., 2011; Cain et al., 2015; Crowe, 2017). Staff working in hospital settings also struggle to maintain healthy weights, have poor dietary habits, and low physical activity levels (Blake et al., 2012; Bogossian et al., 2012). Furthermore, a high proportion (60%) of midwifery nursing staff in Australia, New Zealand, and the United Kingdom (UK) were living with overweight/obesity (Bogossian et al., 2012). In the UK, 40% of healthcare workers have been found to be living with overweight/obesity and partake in sub-optimal behaviours, such as not meeting daily fruit and vegetable consumption nor physical activity recommendations, and regularly consume high fat and high sugary foods (Blake et al., 2012).

Workplace interventions in Australian health service settings to improve the health and wellbeing of hospital staff are scarce and often consist of the Employee Assistance Program, which focus mainly on workplace stress management and serious psychological and mental health difficulties (Arthur, 2000; Bouzikos et al., 2022; Employee Assistance Professional Association of Australasia, 2009). Broader consideration around general health and wellbeing, healthy lifestyle, and physical activity are not usually the remit of employee assistance programs.

In 2018, a rural health service in western Victoria with approximately 800 staff across multiple sites sought to improve the health and wellbeing of its staff and wished to implement and evaluate health promoting interventions across its settings. The health service already had a long-established Employee Assistance Program and was keen to broaden its remit and offer healthy lifestyle opportunities. Prior to designing and implementing intervention strategies, an understanding of staff behaviours and health risks was first required.

However, the health service's plans were significantly disrupted by the COVID-19 pandemic. Therefore, the aim of this study was to examine the health and wellbeing data of healthcare workers in a rural Victorian health service before and during the COVID-19 pandemic and use this knowledge to inform a new baseline of healthcare worker behaviours for the design and implementation of future healthy intervention activities within this workplace setting.

## 2 Methods

### 2.1 Study design

This study was repeat cross-sectional in design, and all staff were invited to participate at both time points of data collection. This study design allows comparisons of health-related outcomes at the macro-level (i.e., workplace setting), despite the potential for different participants being involved at the different time points of data collection (Pan, 2021). This design also allows for the analysis of any aggregate changes that have occurred since the last survey (Steel, 2008). Staff health and wellbeing was an identified strategic priority in the 2018 health service strategic plan (Victorian State Government, 2018). This priority coincided with the establishment of a health and wellbeing committee to develop and implement intervention strategies to improve staff health and wellbeing. Deakin University researchers were invited to provide support in evaluating the effectiveness of these intervention strategies with cross-sectional data collection. The original study plan was to capture data on staff pre-intervention (Time 1) in 2018 and post-intervention (Time 2) in 2020. However, the COVID-19 pandemic significantly disrupted the study design. Rather than post-intervention data being collected in 2020, a shortened survey (due to staff already having significant COVID-related working fatigue and burden) was designed to capture a new baseline for staff. Victoria had experienced significant lockdown periods/stay at home orders before and during data collection Time 2.

### 2.2 Participants

Western District Health Service is a sub-regional provider of healthcare for western Victoria and south east South Australia, with a main campus base hospital and four other sites servicing different towns across two local government areas (Victorian State Government, 2018). It is the major employer in the region, with 887 staff members, of which approximately 566 were equivalent full-time staff and services numerous agricultural communities. In 2020, the majority of staff (53%) were 45 years and over, with 82% identifying as female (Dainty, 2023). In 2018, nurses made up 44% (*n* = 360) of staff at the health service and represented a 43% fulltime equivalent (Dainty, 2023). In 2020, nurses made up 43% (*n* = 385) of staff and 43% fulltime equivalents (Dainty, 2023). There were also contractors that included consultants, rotating medical and clinical trainees, and visiting medical officers. All staff were invited to participate, and there were no exclusion criteria.

### 2.3 Recruitment

A plain language statement and organisational consent form was signed by the chief executive officer of Western District Health Service and returned to researchers at Deakin University. Briefly, this form outlined the study: the risk/benefit of the study; how data would be collected, stored, analysed, and disseminated; and gave permission to staff to participate should they wish to.

A human resources staff member at the health service initially introduced the survey to the staff electronically via a global staff email and verbally at staff and departmental head and team meetings. The email contained the plain language statement/consent form. Hard copies were available upon request. As above, this form briefly outlined the study, the risk/benefit of participating in the study, and how data would be collected, stored, analysed, and disseminated, so participants could make an informed decision on whether to participate or not. Potential participants were given instructions on how to access an Outlook calendar (managed by the human resources staff member) to book appointment times for a health assessment. All appointments made in this Outlook calendar appeared as private for anyone else accessing the calendar, so no one could ascertain who was/was not participating. Participants had the opportunity to speak with a member of the research team via telephone to clarify any questions or concerns prior to data collection. A Deakin University research staff member was also present on the days of data collection for any questions. At Time 2, recruitment was similar; however, data collection was modified. There were no appointments for health assessment – the survey was significantly shorter to reduce burden on staff as it was during the COVID-19 pandemic and was strictly self-reported. A human resources staff member sent out another global invitation to all staff; however, this time, it contained a link to the plain language statement, consent form, and online survey. Whether the invitation email was opened/read by staff was unable to be tracked due to privacy reasons; therefore, a completion rate was calculated in lieu of a response rate by number of completed surveys as numerator over the number of surveys clicked on/started online. The risk of coercion to participate was low given the use of a global email list with direct links to a calendar (Time 1) or survey (Time 2) and the repeated information regarding the voluntary nature of participation, and that there would be no consequence to their relationship with Deakin University and Western District Health Service should staff decide not to participate.

### 2.4 Health assessment

The health assessment at Time 1 consisted of two parts – firstly completing an online survey in the waiting room prior to the appointment; secondly, attending the health assessment appointment whereby nurse practitioners followed a standard protocol to measure anthropometry and blood pressure and conduct the Department of Health Australian Type 2 Diabetes Risk Assessment Tool to assess the participant's risk of type 2 diabetes (Department of Health, 2020). At Time 2, only self-report height and weight were collected via the online survey.

#### 2.4.1 Online survey

Participants were provided with a plain language statement and consent forms on the first page of the online survey using Qualtrics (Provo, Utah, United States of America). At Time 1, hard copies were available in the waiting room for participants to keep if requested. An identification number was given to the participant to enter into the participant survey on the iPad in the waiting room. The participant completed the survey on the iPad, and at the end of the survey there was a “pause” page, and directions for the participant to give the iPad to the health professional when called into a private consultation room for the health assessment. At Time 2, participants were able to access a QR code/link with the online survey via their mobile telephone, iPad, or computer.

Dietary intake was assessed using questions drawn from previously published work. This included number of serves of fruit and vegetables eaten each day, frequency of fast food/takeaways, snacks (e.g., packet potato chips, sweet/savoury biscuits, chocolate, lollies, cakes, muffins, sweet pastries), breakfast consumption, beverage consumption on a usual day, and alcoholic drink frequency (Australian Bureau of Statistics, 2018; Brumby et al., 2013; Kennedy et al., 2013; Victorian Agency for Health Information, 2021).

Physical activity questions included duration and frequency of physical activity, sitting or lying down and sedentary screen-based activity on a usual week day and weekend day, and number of times muscle-strengthening physical activities were conducted (Australian Bureau of Statistics, 2018; Victorian Agency for Health Information, 2021). Physical activity-related questions included time spent walking for >10 min at a time; time spent doing vigorous household chores and gardening; moderate physical activity; and vigorous physical activity (Australian Bureau of Statistics, 2018; Victorian Agency for Health Information, 2021). Other questions included whether there was a television or electronic device with a screen in their bedroom, method of transport to work, and reasons for not being physically active during work hours (including travel to/from work). Specific questions about sleep were extracted from a previous survey tool and included sleep duration and quality, whether their current work schedule allowed individuals to get enough sleep, activities conducted in the hour prior to going to bed, number of days work has been missed due to being too sleepy/problems with physical or mental health, agreement with whether getting enough sleep impacted or affected job performance, and whether they felt they are getting enough sleep (Adams et al., 2016).

Health and wellbeing were assessed using the Kessler 10 (Kessler et al., 2003), a set of 10 questions examining emotional states using a 5-level response scale. At Time 1, self-perceived quality of health and bodily pain, satisfaction with weight and size, and whether the individual had tried to lose weight in the last 12 months was also assessed (Brumby et al., 2012; Victorian Agency for Health Information, 2021).

Demographic information collected included sex, age category (18–24; 25–34; 35–44; 45–54; 55–64; 65 or more), postcode, primary employment status in the last 3 months at the health service (working <one job, full-time, part-time, casual, student, volunteer, other), work schedule (regular day/evening/night shifts, rotating shifts, other), Indigenous status, and country of birth. Smoking status and frequency were also collected (Victorian Agency for Health Information, 2021).

The survey took approximately 10–15 min to complete.

#### 2.4.2 Anthropometry

##### 2.4.2.1 Time 1

Anthropometric measurements (height, weight, waist circumference) and blood pressure were collected by trained health practitioners following standard measurement procedures in a private consultation room. Measurements were recorded directly into an electronic data collection form by the health practitioners on an iPad. All equipment was calibrated prior to use.

Participants were asked to remove any heavy outer clothing, shoes, belts, or jewellery and empty their pockets. Measurements were taken over one light layer of clothing. Height and weight were collected. Height was measured to the closest 0.1 cm. If the two measurements disagreed by >0.5 cm, a third was taken. Waist circumference was measured using a standard flexible measuring tape on the umbilical line. Two measurements were taken to the 0.1 cm. If the two measurements disagreed by >0.3 cm, a third was taken. The average of the two closest measurements was used in analysis. Body weight was measured using a TANITA DC430MA Body Composition Scale (Wedderburn, 2020), which was set up on a hard tiled surface (not carpet), and the equipment was centred according to the inbuilt levelling gauge. Two measurements were taken to the nearest 0.1 kg, and if there was a disagreement by >0.1 kg, a third was taken.

Blood pressure was measured using an Omron automatic blood pressure monitor (Model IA1B) whilst participants were seated with feet flat on the floor. The cuff was applied directly to the skin on the left arm. Two minutes rest was allowed between each measurement. Participants were offered a free coffee at the on-site food retailer for participating. They also received an individual copy of their results via mail, which included recommendations to contact their local general practitioner should they have concerns, and also the local contact for the Employee Assistance Program was made available.

##### 2.4.2.2 Time 2

Participants were asked to self-report height (centimetres [cms]) and weight (kilogram [kgs]) during the online survey.

### 2.5 Statistical analysis

Descriptive statistics were conducted on all variables. Statistical significance was determined by chi-square or independent *t*-test where appropriate with a *p* < 0.05 considered statistically significant. Data was examined against Australian dietary guidelines for fruit and vegetable consumption (National Health and Medical Research Council, 2013), National Health and Medical Research Council (2021) guidelines for alcohol consumption, and the Australian physical activity guidelines (The Department of Health and Aged Care, 2021). The Australian physical activity guidelines for 18–64 years include accumulating 150–300 min of moderate or 75–150 min of vigorous-intensity physical activity per week and doing muscle-strengthening activities on at least 2 days per week (The Department of Health and Aged Care, 2021). The Kessler 10 level of psychological distress was calculated by assigning points per response category as per standard protocol (Kessler et al., 2003). Responses were coded as follows: none of the time (1 point), a little of the time (2 points), some of the time (3 points), most of the time (4 points), all of the time (5 points), and then summarized for each of the 10 questions to generate the following distress categories: a score of <20 were likely to be well; ≥20–≤24 were likely to have a mild mental disorder; ≥25 - ≤29 were likely to have a moderate mental disorder; and ≥30 were likely to have a severe mental disorder (Kessler et al., 2003). Similarly, the responses to the questions in the Australian Type 2 Diabetes Risk Assessment Tool were also scored with points as previously described (Department of Health, 2020). The points were then totalled for the 10 questions with a score of 5 or less indicating low risk; 6–11 indicating intermediate risk, and 12 or more indicating high risk of developing type 2 diabetes in the next 5 years. Body mass index was used to categorise weight status categories according to World Health Organization (2010).

### 2.6 Ethics approval

Approval to conduct this study was given by the Deakin University Human Ethics Advisory Group (HEAG-H 138_2018).

## 3 Results

Of the 800 staff, 184 staff participated from three sites at Time 1 (100% completion rate) and 87 at Time 2 (73% completion rate). Note that 22 individuals participated at both Time 1 and Time 2. The demographic characteristics of the participants are displayed in Table 1. In summary, at both timepoints, the majority of participants were female, more than half were over the age of 45 years , and most were born in Australia. At Time 1 only;2% of participants identified as Indigenous . Approximately half of the participants worked part-time and over a third full-time, and most worked regular day shifts in the past 3 months. A small proportion were currently smoking. There were no significant differences in demographic characteristics, except for work schedule (e.g., Time 2 had a higher proportion of staff with regular night shifts) and smoking status (e.g., Time 2 had a higher proportion of non-smokers and lower proportion of ex-smokers).

**Table 1:** Demographic characteristics of participating staff Time 1 and Time 2.

	**Time 1 2018** ***n*=184**		**Time 2 2020** ***n*=87**		**Time 1vs Time 2**
**Characteristic**	***n***	**%**	***n***	**%**	*p* value*
**Sex**					0.708
Female	155	84	73	84	
Male	29	16	14	16	
**Age category**					0.234
18–24	13	7	2	3	
25–34	30	16	21	24	
35–44	26	14	17	20	
45–54	60	33	30	35	
55–64	49	27	15	17	
65 or more	6	3	1	1	
**Country of birth**					0.207
Australia/other	176	96	66	92	
Asia (incl. Indian sub-continent, Middle East, North Africa, Southern Europe)	8	4	13	18	
**Indigenous status**					
Yes	3	2	0	0	
**Employment status at the health service in the last 3 months**					0.527
Working more than one job	11	6	3	4	
Working full-time	71	39	26	36	
Working part-time	91	49	39	54	
Casual	5	3	4	6	
A student	1	1	0	0	
Other (specify)	5	3	0	0	
**Work schedule in the last 3 months**					0.025*
Regular day shifts	128	70	47	65	
Regular evening shifts	3	2	0	0	
Regular night shifts	0	0	3	4	
Rotating shifts	44	24	21	29	
Other (specify)	9	5	1	1	
**Smoking status**					0.041*
Current	16	9	6	7	
Ex-smoker	54	29	14	16	
Non-smoker	114	62	67	77	
**Smoking frequency**					0.237
I smoke daily	12	7	5	6	
I smoke occasionally	6	3	2	2	
I don't smoke now but I used to	59	32	17	20	
I've tried a few times but never smoke	27	15	17	20	
I've never smoked	80	43	46	53	

*n:* number of participants; *p value by chi-square whereby *p* < 0.05 was considered statistically significant; note missing data for some variables.

### 3.1 Dietary and physical activity-related behaviours

Key dietary and physical activity-related behaviours are displayed in Table 2. Regarding dietary guidelines, at Time 2, a slightly higher proportion of staff met fruit guidelines, but a lower proportion met vegetable guidelines of >5 serves. At Time 2, there was a significant change in fast food/takeaway frequency, with a higher proportion of participants reporting consumption once per week and 2-3 times a week, compared to Time 1. Packaged snack consumption was consumed frequently at both timepoints. The proportion of participants that did not have breakfast on the day of the survey increased at Time 2.

**Table 2:** Dietary-related behaviours of participants who completed the survey.

**Characteristic**	**Time 1 2018**					**Time 2 2020**					**Time 1 vs Time 2**
	***n***		**%**			***n***			**%**		*p* value*
**Serves of fruit**											0.728
Do not eat fruit	7		4			5			6		
<2 serves	101		55			45			52		
≥2 serves	76		41			37			42		
**Serves of vegetables**											0.571
≤1 serve	9		5			7			8		
1-2 serves	66		36			26			30		
3-4 serves	76		41			43			49		
5 serves	24		13			9			10		
5.5 serves	4		2			1			1		
≥6 serves	5		3			1			1		
**Fast food/takeaway frequency**											0.018*
Never	12		7			3			3		
Once a month or less	66		36			23			26		
2-3 times per month	49		27			17			20		
Once per week	48		26			34			39		
2-3 times a week	7		4			10			11		
Most days	2		1			0			0		
**Packaged snack consumption**											0.191
Never	5		3			1			1		
Once a month	17		9			5			6		
2-3 times a month	17		9			15			17		
Once a week	44		24			21			24		
2-3 times a week	68		37			24			28		
Most days	33		18			21			24		
**Breakfast**											0.091
Yes	148		80			62			71		
	**Time 1 2018**					**Time 2 2020**					**Time 1 vs Time 2**
Beverages consumed on a usual day (ml)*	***n***	**Mean ml**			**SE**	***n***	**Mean ml**			**SE**	*p* value*
Water	164	1142.24			51.92	85	1016.47			86.05	0.187
Bottled water	39	688.46			79.91	26	755.77			102.58	0.603
Cordial	12	445.83			68.94	8	393.75			101.52	0.664
Sugar sweetened beverages	4	312.5			36.08	8	262.5			30.62	0.346
Diet beverages	9	391.67			83.12	15	512.00			75.17	0.314
Tea	100	595.9			34.04	45	513.33			47.63	0.171
Coffee	118	538.86			27.00	63	489.68			35.00	0.276
Flavoured milk	8	368.75			68.75	3	175.00			101.04	0.167
Non-flavoured soda/mineral water	20	315.00			28.81	15	353.33			33.62	0.392
Combined cordial/sugar sweetened beverages/flavoured milk	34	452.94			48.87	16	287.50			56.69	0.047*
	**Time 1 2018**					**Time 2 2020**					**Time 1 vs Time 2**
	***n***			**%**		***n***		**%**			*p* value*
**Have you had an alcoholic drink of any kind in the last 12 months?**											
Yes	163			89		73		84			0.284
**In the last 12 months, how often did you have an alcoholic drink of any kind?**											0.005*
Less than 1 day a month	24			15		26		36			
About 1 day a month	12			7		5		7			
2-3 days a month	42			26		15		21			
1-2 days a month	52			32		10		14			
3-4 days a week	22			14		13		18			
5-6 days a week	7			4		2		3			
Every day	3			2		1		1			
Don't know	1			1		0		0			
**How often do you have more than 4 standard drinks on a single occasion? (risk of alcohol-related injury from a single occasion of drinking)**											0.049*
Never	46			28		33		45			
Less than monthly	52			32		22		30			
Monthly	40			25		12		16			
Weekly	25			15		6		8			
**How often do you have more than 2 standard drinks on a single occasion? (lifetime risk of alcohol-related harm)**											0.025*
Never	18			11		16		22			
Less than monthly	49			30		30		41			
Monthly	49			30		15		21			
Weekly	45			28		12		16			
Daily	2			1		0		0			
**How many standard drinks do you have on a typical day when drinking?**											0.416
<1	12			7		9		12			
1	40			25		24		33			
2	51			31		14		19			
3	14			9		6		8			
4	17			10		11		15			
5	9			6		3		4			
6	10			6		4		5			
7	2			1		0		0			
8	4			2		0		0			
≥9	4			2		2		3			

*n:* number of participants; SE: standard error; Mean ml of beverages calculated for each beverage type based upon the number of participants that had reported drinking any kind of beverage on a usual day (i.e., mean calculation did not include participants who reported not drinking that type of beverage on a usual day). *p value by chi-square or independent *t*-test where appropriate, whereby *p* < 0.05 was considered statistically significant.

Water, coffee, and tea were the most popular beverages consumed (Table 2); however, almost a fifth of participants consumed cordial/sugar sweetened beverages/flavoured milk in a usual day. Regarding alcohol consumption, almost a fifth reported drinking at least 3 days or more per week, and 40% reported drinking at high-risk levels of more than four standards drinks in any occasion at least monthly at Time 1. In the last 12 months, a higher proportion of staff reported drinking less frequently (less than 1 day a month; 15% Time 1 vs 36% Time 2). The proportion of staff who drank alcohol weekly and monthly at a level that increased their lifetime risk of alcohol-related harm (>two standard drinks on a single occasion) or at a level that increased their risk of alcohol-related injury from a single occasion of drinking (>four standard drinks on a single occasion) was significantly reduced at Time 2. On a typical day when drinking, two thirds reported drinking two standard drinks or fewer at both timepoints.

Less than half of the staff met the physical activity guidelines at both timepoints (Table 3). Almost a fifth of staff at both time points reported not doing 30 min of moderate and/or vigorous physical activity per day (16% Time 1 vs 18% Time 2, *p* value 0.345, data not shown). Just under 50% of staff at both time points participated in sufficient physical activity per week. The majority of participants used a car to get to/from work. At Time 1, the top five reasons for not being physically active during work hours, including travel to and from work, included already exercising out of work hours (28%), living too far from work (18%), other (18%, examples included already active, gait issues, asthma, haven't found a gym to sign up to yet, walk at lunch time, lack of supportive environment [i.e., meetings always sedentary, lunch time meeting, role, sedentary work]), not having enough time (9%), and not enough flexible time in work hours (7.6%) (data not shown, and not collected at Time 2).

**Table 3:** Physical activity-related behaviours of participants who completed the survey.

	**Time 1 2018**				**Time 2 2020**				**Time 1 vs Time 2**
	***n***		**%**		***n***		**%**		*p* value*
**Sufficient physical activity (75 min VPA OR 150 min MVPA) + 2 sessions of strength training/ week)**									
Yes	87		47		32		44		0.617
**TV or electronic device with a screen in your bedroom**									
Yes	83		45		35		46		0.019
**Usual way to get to work**									
Walk	19		10		5		7		0.637
Car	161		88		68		90		
Motorbike/motorized scooter	0		0		0		0		
Bicycle/scooter	2		1		1		1		
Bus	0		0						
Other (taxi, multiple ways)	2		1		2		3		
**Time spent doing physical activity or sedentary activity in a usual day – weekday (mins)**									
**Weekday physical activity**	***n***	**Mean (mins)**		***SE***	***n***	**Mean (mins)**		**SE**	*p* value*
Moderate	165	254.23		18.01	71	164.17		18.01	0.003*
Vigorous	163	169.87		13.08	60	153.68		22.08	0.524
Sedentary	179	344.67		19.95	72	350.86		37.41	0.875
Moderate/vigorous physical activity	184	528.95		35.09	87	345.95		40.28	0.002*
**Time spent doing physical activity or sedentary activity in a usual day – weekend day (mins)**									
**Weekend physical activity**	***n***	**Mean (mins)**		***SE***	***n***	**Mean (mins)**		**SE**	*p* value*
Moderate	154	224.60		20.44	61	148.36		17.05	0.027*
Vigorous	157	206.54		15.93	55	199.54		28.60	0.826
Sedentary	154	347.40		20.27	61	336.31		30.46	0.768
Moderate/vigorous	169	588.44		43.58	64	484.37		60.8	0.195

*n:* number of participants; SE: standard error; moderate physical activity is the total of time walked for at least 10 min at a time plus moderate physical activity for fitness, recreation or sport; vigorous physical activity is the total time doing vigorous household chores and gardening plus vigorous physical activity for fitness, recreation or sport that cause the participant to breathe harder or puff and pant; sedentary is total time sitting or lying down for activities outside of work plus sedentary screen-based activity *p value by chi-square or independent *t*-test where appropriate, whereby *p* < 0.05 was considered statistically significant.

### 3.2 Sleep

Sleep behaviours were described (Table 4). On average, participants reported sleeping almost 7 h on a work/weekday, and 7.7 h on a non-work/weekend day at both time points. At Time 1, 77% of participants thought that their current work schedule or typical weekday routine allowed for enough sleep (data not shown, data not collected Time 2). At both time points, almost a quarter of staff did work relating to their job within an hour before going to bed. A large majority were using screens (television or internet) or reading within an hour before going to bed every night/almost every night. More than two thirds agreed that not getting enough sleep affected their job performance at Time 1 (data not shown), and 84% rated their sleep average or better at Time 1, but only 71% at Time 2 (*p* = 0.231). At Time 1, 22% staff reported feeling like they never/almost never got enough sleep, but at Time 2, 32% felt this way (*p* = 0.267).

**Table 4:** Sleep behaviours of participants who completed the survey.

	**Time 1 2018**			**Time 2 2020**				**Time 1 vs Time 2**
**Characteristic**	***n***	**Mean**	**SE**	*n*	**Mean**		**SE**	*p* value*
**Sleep (mins)**								0.779
Workdays or weekday	183	417.87	4.77	70	420.43		7.81	
Non-workdays or weekend	180	459.83	4.97	70	465.86		8.43	
	**Time 1 2018**			**Time 2 2020**				**Time 1 vs Time 2**
	*n*	%		*n*		%		*p* value*
**In the past month did you do work relating to your job within an hour of going to bed?**								0.844
Every night or almost every night	10	6		4		6		
A few nights a week	33	20		12		17		
A few nights a month	22	13		9		13		
Rarely	48	28		17		24		
Never	56	33		29		41		
**In the past month did you watch TV within an hour of going to bed?**								0.024*
Every night or almost every night	107	59		40		56		
A few nights a week	45	25		20		28		
A few nights a month	12	7		3		4		
Rarely	14	8		2		3		
Never	3	2		7		10		
**In the past month did you listen to the radio or music within an hour of going to bed?**								0.577
Every night or almost every night	14	9		10		14		
A few nights a week	30	18		9		13		
A few nights a month	26	16		14		19		
Rarely	40	24		16		22		
Never	52	32		23		32		
**In the past month were you on the internet within an hour of going to bed?**								0.728
Every night or almost every night	77	45		32		44		
A few nights a week	49	28		19		26		
A few nights a month	16	9		10		14		
Rarely	21	12		9		13		
Never	10	6		2		3		
**In the past month did you read within an hour of going to bed?**								0.333
Every night or almost every night	46	26		14		19		
A few nights a week	33	19		18		25		
A few nights a month	32	18		14		19		
Rarely	48	27		15		21		
Never	16	9		11		15		
**Overall, how well do you think you sleep?**								
Very good	32	17		11		15		0.231
Fairly good	62	34		18		24		
Average (not good but not bad)	60	33		24		32		
Fairly bad	24	13		17		23		
Very bad	6	3		4		5		
**On average, how often do you feel you get enough sleep?**								
Never	10	5		6		8		0.199
Almost never	31	17		18		24		
Sometimes	62	34		23		31		
Often	34	19		17		23		
Almost always	46	25		10		14		

*N:* total number of participants;*n:* subgroup number of participants; SE: standard error; *p value by chi-square or independent *t*-test where appropriate, whereby *p* < 0.05 was considered statistically significant.

### 3.3 Health and wellbeing

Health and wellbeing were measured by self-report and validated tools (Supplementary Material Tables 1, 2). The majority of participants described their health as good/very good/excellent. However, a third of participants reported to have severe/very severe bodily pain, felt their health impacted their normal activities in the past 4 weeks, and were unhappy/very unhappy with their weight and size. Two thirds of participants had tried to lose weight in the last 12 months. Using the Kessler 10 scale, at Time 1, 42% of participants were likely to be suffering moderate to very high levels of psychological stress, which increased to 59% at Time 2 (*p* = 0.012). When examining risk of developing type 2 diabetes in the next 5 years, 28% were thought to have low, 39% intermediate, and 33% high risk. None of this data was collected at Time 2.

### 3.4 Anthropometric measurements

Health professionals collected anthropometric measurements at Time 1 (Table 5). Due to the COVID-19 pandemic, height and weight was self-reported at Time 2 (Table 5). Looking at the classifications of body mass index more closely, roughly a third of participants were in each of the healthy weight, overweight, and obese categories; however, there was 6% more staff in the obesity category at Time 2 (*p* = 0.637). Examining dichotomous body mass index categories, two thirds were classified as living with overweight or obesity at both time points. At Time 1, a third of participants were considered hypertensive, according to their blood pressure (data not shown).

**Table 5:** Anthropometric measurements of participants.

	**Time 1 2018**			Time 2 2020*			**Time 1 vs Time 2**
	***n***	**Mean**	**SD**	***n***	**Mean**	**SD**	*p* value*
Height cms	182	166.49	7.61	69	165.22	8.07	0.245
Weight kgs	180	77.79	16.87	67	78.75	17	0.694
Waist cms	168	89.05	14.10	–	–	–	
Waist (females only) cms	151	87.54	13.93	–	–	–	
Body mass index	178	27.95	5.37	66	29.29	6.977	0.112
**Weight status: 4 categories**							
Underweight	2	1		0	0		0.637
Healthy weight	62	35		22	33		
Pre-obesity (overweight)	56	32		18	27		
Obesity (class I-III)	58	33		26	39		
**Weight status: 2 categories**							
Underweight/normal weight	64	36		22	33		0.703
Pre-obesity/obesity (class I-III)	114	64		44	67		

*n:* number of participants; SD: standard deviation; cms: centimetres; kgs: kilograms. *p value by chi-square or independent *t*-test where appropriate, whereby *p* < 0.05 was considered statistically significant; due to COVID-19, Time 2 anthropometric measurements were self-report, and blood pressure was unable to be measured.

Staff reported that prior to COVID-19, only 7% worked from home, but all 100% agreed they worked more from home during COVID-19 (Supplementary Material Table 3). None of the staff surveyed had contracted COVID-19 at the time of surveying. When examining the impacts of COVID-19 on various aspects of staff lives, only a small proportion of staff reported positive impacts, with the majority reporting either no impact or negative impacts (Supplementary Material Table 4). A higher proportion of staff reported the following areas/aspects were negatively impacted by COVID-19: stress (78%), social connection (67%), and friend relationships (55%). Over two thirds reported COVID-19 to have no impact on studies (63%) or employment (62%), and around a fifth reported a positive impact on finances (22%) and exercise (18%). Childcare, homeschooling, elderly, and family infection were the top aspects staff reported to be very/extremely stressed about.

## 4 Discussion

### 4.1 Principal findings

This large repeat cross-sectional study of behaviours in healthcare workers of a rural health service in Victoria revealed staff often had less than optimal dietary and physical activity-related behaviours. Two thirds of staff were classified as living with overweight/obesity and had risk factors for future chronic disease (e.g., smoking, elevated waist circumference). Fruit, vegetable, and sugary beverage consumption was not in alignment with Australian dietary guidelines, and less than half of staff met the Australian physical activity guidelines at both timepoints. Alcohol consumption behaviours at Time 2 had improved. Some sleep routines were not in alignment with best practice sleep hygiene (i.e., high use of screens within an hour of going to bed). At Time 1, the risk of developing type 2 diabetes in the next 5 years was high for a third of staff. COVID-19 largely had a negative impact and increased stress of staff in areas such as childcare, homeschooling, elderly, and family infection; however, staff did not report a significant impact on diet or physical activity. The finding regarding a significant increase in staff at Time 2 reporting moderate to very high levels of psychological stress should be the first focus of future intervention as we exit the intensity of the COVID-19 pandemic.

### 4.2 Diet

The current dietary and physical activity-related findings from this sample of healthcare workers in rural Victoria are reflective of previous international and national studies and have similarities with the general Australian population. A large proportion of staff in this sample did not meet Australian dietary guidelines for vegetables or fruit, and a fifth of staff consumed cordial/sugar sweetened beverages/flavoured milk in a usual day. Despite compliance with dietary guidelines being low, it was similar to a small study on Queensland nurses where 62% met fruit and 15% met vegetable guidelines in 51 nurses (Happell et al., 2014). Interestingly, compliance with these dietary guidelines in this study population of healthcare workers was higher compared to the 2019 Victorian Population Health Survey, where 7% met vegetables and 41% met fruit guidelines (Victorian Agency for Health Information, 2021). Whilst international guidelines for vegetable and fruit intake may differ, surveys of healthcare workers have also revealed low compliance with relevant guidelines in the UK (Malik et al., 2014; Mittal et al., 2018); Scotland (Schneider et al., 2019) and Israel (Kagan et al., 2021).

Breakfast skipping was reported by 20–30% staff surveyed, and, whilst there is limited data in healthcare workers to compare this to, more than half (56%) of Israel nurses have been reported to skip breakfast or lunch every day or almost every day (Kagan et al., 2021). Research regarding shift working nurses revealed high workloads and demands during shifts as reasons for skipping meals and an inability to drink enough water (Gifkins et al., 2018). Adequate water consumption to prevent dehydration during work is vital, and a potential yet significant occupational hazard for staff (Gifkins et al., 2018). At both time points, approximately two cups of tea and coffee were consumed by staff surveyed. Whilst analysis of caffeine consumption was not stratified by shift type due to the small sample size, it has been suggested that nurses on night shifts do increase caffeine consumption to overcome fatigue and provide an energy boost (Gifkins et al., 2018).

The level of alcohol consumption had reduced at Time 2 in this study, with consumption levels slightly lower than the general Victorian population data (which was collected pre-COVID-19). Sixteen percent of staff reported weekly lifetime risk of alcohol-related harm (>two standard drinks on a single occasion) in comparison to 21% of the general population (Victorian Agency for Health Information, 2021). Regarding risk of alcohol-related injury from a single occasion of drinking (i.e., having >four standard drinks on a single occasion), 8% staff reported this to occur weekly compared to 12% of the general population (Victorian Agency for Health Information, 2021). Note that 40% of healthcare workers in this study consumed alcohol at this rate at least monthly. It is important to consider the low percentage of male staff that participated in this current study, and comparing to a general population may not be valid. Whilst some evidence has suggested nurses drink alcohol to wind down and relax after work (Gifkins et al., 2018), there appears to be little evidence within this sample of healthcare staff.

### 4.3 Physical activity

Less than half of the staff met the physical activity guidelines at both timepoints. This is similar to 51% of the Victorian state population data (Victorian Agency for Health Information, 2021) and 55% of the national Australian population (Australian Institute of Health and Welfare, 2022). An additional study conducted in regional Queensland in nurses using different physical activity guidelines (i.e., without the two sessions of muscle-strengthening activities per week), reported almost two thirds of participants met the recommended levels of both moderate and vigorous physical activity (Happell et al., 2014). These findings are similar to international studies with UK studies showing that just under half (48.6%) did not partake in any physical activity on most days of the week for 30 min or more (Malik et al., 2014), and only 56% of staff undertook moderate or vigorous physical activity (Mittal et al., 2018). About a fifth of UK staff undertook muscle-strengthening exercises and yoga (Mittal et al., 2018), and a Scottish study reported 46% nurses not meeting physical activity guidelines (Schneider et al., 2019).

Key barriers to physical activity during work hours reported by this sample of healthcare workers at Time 1 included already exercising out of work hours, living too far from work, other (e.g., already active, health issues, lack of supportive environment), not having enough time, and not enough flexible time in work hours. These barriers are similar to those already reported internationally: not enough time (Malik et al., 2014; Mittal et al., 2018), lack of motivation, physical disability, lack of facility at work or near home, other reasons (tiredness, child care issues, and expense) (Mittal et al., 2018), being too tired, cannot afford it, and having no motivation (Malik et al., 2014). A UK study in student nurses also found time, cost, and tiredness to be factors influencing engagement in physical activity (Blake et al., 2011).

### 4.4 Sleep

Sleep is an integral part of good health and wellbeing. Adequate recovery after shifts by relaxing, resting, and sleeping is important (Gifkins et al., 2018). Similar to national data (Adams et al., 2016, 2017), on average staff reported sleeping almost 7 h. Sleep hygiene behaviours reported by healthcare staff in this survey were less than optimal in many cases but similar to national sleep data highlighting the effect of the ‘24/7 society’ (Adams et al., 2016), with a high number of staff reporting screen time and work an hour before going to bed, and many staff reported feeling like they never/almost never got enough sleep. Shortened sleep times may carry increased risk of poor health, such as poorer immune function, obesity, type 2 diabetes, and heart disease and may have substantial economic and social costs due to accidents and decreased workplace performance and productivity (e.g., absenteeism and presenteeism) (Adams et al., 2016, 2017). This productivity concept was supported by healthcare staff in the current survey, with more than two thirds agreeing that not getting enough sleep affected their job performance. Lifestyle choices prior to going to bed may impact sleep habits and subsequently impact not only sleep but daytime performance (Adams et al., 2016, 2017). Sleep should be more of a community priority (Adams et al., 2016, 2017).

### 4.5 Other risk factors for chronic disease

The high prevalence of overweight and obesity in the current sample (67% at Time 2) was similar to 62% previously found in Australian nurses (Bogossian et al., 2012). This is also similar to the 67% prevalence in national Australian data (Australian Bureau of Statistics, 2018). In the UK, findings have been conflicting, with 39% of staff classified as living with overweight/obesity in one study (Malik et al., 2014), and half either overweight or obese in another study (Mittal et al., 2018). A lower prevalence living with overweight and obesity (27%) has been found in pre-registration nurses (Blake et al., 2011), and it could be hypothesised that age of sample (mean age was 24 years old) and timing of data collection (in 2011) may have impacted the lower prevalence reported.

When examining risk of developing type 2 diabetes in the next 5 years at Time 1, the level of risk was not surprising given the low compliance to dietary and physical activity guidelines, which are key risk factors for metabolic disease. Furthermore, at Time 1, the average waist circumference for females indicated increased risk of disease and was very close to the substantially increased risk cutoff point. Overall, these findings were similar to the national average for females in 2015 (Australian Bureau of Statistics, 2015).

Smoking in this sample of healthcare staff was lower than the Victorian general population - 6% Time 1 and 7% Time 2 vs 12% Victorian population (Victorian Agency for Health Information, 2021). This is also lower than the international smoking prevalence reported by healthcare staff in UK (19% nurses (Blake et al., 2011; Malik et al., 2014), and Scotland (17% nurses smoked, 24% staff overall (Schneider et al., 2019)). The health service has been smoke-free since 2008, and smoking rates are likely to reflect this.

### 4.6 Impact of COVID-19 on the health and wellbeing of healthcare staff

At Time 1, 42% of participants were likely to be suffering moderate to very high levels of psychological stress, which significantly increased to 59% at Time 2. This is higher than the Victorian population average of 47% (Victorian Agency for Health Information, 2021); however, the Victorian state data were collected prior to COVID-19. A Victorian wellbeing impact study used a shortened version of Kessler 10 (the Kessler 6) and found high psychological distress in 17% of the participants (Victorian Health Promotion Foundation, 2020). This is slightly lower than the 20% categorised as high reported by staff in this survey.

The impact of COVID-19 on healthcare staff has been well documented, with staff reporting high levels of fear-related symptoms, poor quality of life, reduced wellbeing and acute stress (Dragioti et al., 2022). Time 2 data collection occurred in 2020 during the COVID-19 pandemic when there were lengthy lockdown periods and changing restrictions (e.g., regarding exercise and social interactions, travel distance, schooling, retail, eating out, and public gatherings). In the rural healthcare setting where the current study was based, a higher proportion of staff was likely to have a mental health issue at Time 2 during COVID-19 compared to Time 1 (though not statistically significant). A recent study of 702 attendees at south west Victoria COVID-19 screening clinics showed 22% of attendees experienced high to very high levels of psychological distress using the Kessler 10 (Rahman et al., 2022). Of particular note is that 43% of these attendees were essential workers (Rahman et al., 2022). Other studies in Australian metropolitan health services have also shown an increases in mental health symptoms and psychological distress associated with the COVID-19 pandemic in healthcare workers (Dobson et al., 2021; Holton et al., 2021a, 2021b; Smallwood et al., 2021); however, direct comparison to the current study isn't possible due to the different psychological distress tools utilised.

A higher proportion of healthcare staff in this survey reported the following areas/aspects to be negatively impacted by COVID-19: stress (78%), social connection (67%), and friend relationships (55%). Over two thirds reported COVID-19 to have had no impact on studies (63%) or employment (62%), and around a fifth reported a positive impact on finances (22%) and exercise (18%). Childcare, homeschooling, elderly, and family infection were the top aspects staff reported to be very/extremely stressed about.

These data are in alignment with a plethora of data collected from healthcare and hospital staff during COVID-19 pandemic. Metropolitan healthcare staff reported negative impacts such as: personal lives, including potentially infecting family members with COVID-19 and their own health and wellbeing, patient care, use and effect of personal protective equipment (e.g., headaches, dehydration, impact on communication), stress related to redeployment, financial concerns, pressure related to remote learning for school-aged children, and uncertainty about the possible impact of COVID-19 on pregnancy (Holton et al., 2021a, 2021b). Another Australian study of metropolitan Victorian healthcare workers revealed seven key themes influencing staff anxiety (Digby et al., 2021). This included: worrying about patient care, changed working conditions, working in the changed hospital environment, impact of the pandemic, personal isolation and uncertainty, leadership and management, and additional support needed for staff (Digby et al., 2021). Despite the many negative impacts described, some positive impacts of COVID-19 were also reported – the learning experience, the increased awareness and knowledge of disease control, an increased sense of togetherness, and cooperation among staff (Holton et al., 2021a, 2021b). Whilst external factors outside of the control of the health service, such as lockdown restrictions, social disconnection, and media coverage may have influenced mental health of healthcare staff, the occupational barriers to a healthy lifestyle reported both in the Time 1 survey and reflected in the health assessments must be addressed (Smallwood et al., 2021). Finally, weight gain, increased stress, and increased alcohol consumption were consistent with other studies conducted during COVID**-**19 (Calina et al., 2021; Grieger et al., 2022; Zeigler, 2021).

### 4.7 Implications

Whilst it might be expected that healthcare staff should have healthier lifestyles compare to the general population given their education and role in influencing health outcomes, this is often not the case (Schneider et al., 2019). Nurses have high health literacy, yet are suboptimal with their own health; and work-related stress, fatigue, and long working hours (Kagan et al., 2021), as well as the desire to care for others might contribute (Dill et al., 2016). Shift work to allow uninterrupted patient care is integral to healthcare staff work practices and it is not realistic/viable to abolish (Gifkins et al., 2018). Complex relationships between food and fatigue have been reported – nurses feeling too tired to exercise yet engaging in unhealthy eating behaviours to prevent fatigue (i.e., sugary snacks and caffeine), which then often unintentionally contribute to it (Gifkins et al., 2018). More broadly, geographic locale also influences health with rural dwellers facing health inequities compared to their urban counterparts; for example, there is a higher prevalence of obese individuals in rural areas compared to urban areas (Barnidge et al., 2013; Cleland et al., 2010). Recently, it has been suggested that rural Australians face a triple disadvantage that leads to poorer health outcomes. These are related to 1. Social determinants of health; 2. Poor service availability; 3. Higher cost of access and higher cost of delivery (Nous Group on behalf of National Rural Health Alliance, 2023). Access to quality, fresh, affordable, and healthy foods is disproportionate compared to those living in an urban area; along with other challenges, such as access to health services, telecommunications/internet, access to fluoridated water (risk of dental caries), and the impact of other social determinants of health, such as education and income (Nous Group on behalf of National Rural Health Alliance, 2023). Rural Australians also face greater vulnerability to climate change and its subsequent impact on water resources, ecosystems, health, infrastructure, and the economy (Nous Group on behalf of National Rural Health Alliance, 2023).

Despite society's ambition to live in “covid-normal” times (Australian Health Protection Principal Committee, 2021), the ripple effects of the pandemic continue today, with staff exhaustion, staff illness, family caring responsibilities, furloughing, and continued staff shortages. The extreme and sustained stress experienced by healthcare workers was recognised by the Victorian government's healthcare worker winter retention and surge payment to thank healthcare workers in public health services and ambulance in Victoria (Victorian Government, 2022). Nurses have been forever altered by working during COVID-19 (Maben et al., 2022), and the need to restore wellbeing is recognised not only in Australia but globally (Holton et al., 2021a, 2021b; Maben et al., 2022; Ohta et al., 2021). Considerable staff shortages, change in work practices, wearing of personal protective equipment, and unpredictable anxiety all impact staff in prioritising healthy lifestyles, such as healthy eating, adequate sleep, and physical activity.

Healthcare settings can implement strategies to encourage healthier environments for working staff. A range of opportunities have been suggested in the literature including: on site access to healthier food (catering and food provision in hospital cafes and canteens), on site exercise facilities, education and awareness-raising, breaks for light physical activity/stretches, health standards for vending machines, adequate meal/drink breaks, provision of free healthy snacks for staff (particularly for those on night shift), and psychological support (Digby et al., 2021; Kagan et al., 2021; Mittal et al., 2018; Schneider et al., 2019; Williams, 2017). Organisational leadership and managerial support (Digby et al., 2021; Smallwood et al., 2021) is also crucial. Strategies to support staff sleep hygiene would also be beneficial. A recent systematic review examining workplace interventions in hospital settings to improve health of staff has revealed multicomponent strategies, financial incentives, and motivational strategies to be most effective (Worley et al., 2022).

Following the response from Time 1, the health service, with leadership from the chief executive officer, board, and executive, made major changes to the health service café and now offers only 'green' food choices with all items having been assessed by the Healthy Eating Advisory Service (Allen, 2018; Healthy Eating Advisory Service, 2023a, 2023b). This practice has continued and led the way for other health services to follow suit. However, due to the COVID-19 pandemic, the benefit of this approach has been difficult to fully assess.

Improving the workplace environment for healthcare staff benefits not only the staff themselves but also the actual healthcare system (Kagan et al., 2021). Implementing system level initiatives that reduce physical and mental stress, complemented with interventions on individual behaviours, is required; particularly in a time of global staff shortages (Schneider et al., 2019). It is important to continue monitoring the healthcare workforce to support their optimal health long-term. It is also important to intervene early – the promotion of health should begin in undergraduate curricula (Schneider et al., 2019) and not just for nurses but all health-related professionals more broadly.

Healthcare staff, particularly nurses, are the front line and foundation of the healthcare system (Williams, 2017). Health services need to extend their remit to assist staff to maintain or improve their health, particularly with expected growing patient needs (Williams, 2017) and the unstable and stressed climate in which they are currently working. A healthy workplace culture will foster staff retention and future recruitment of staff (Pascoe et al., 2022). Considering the health inequities rural dwellers face, maintaining a healthy and sustainable rural health workforce is vital to address ever-increasing population needs.

## 5 Strengths and limitations

Strengths of this study include the repeated cross-sectional design, which allows consideration of change at the aggregate/population level (Rafferty et al., 2015), therefore providing insight into the health and wellbeing of health service staff in a rural Victorian health service. These data provide an update on what behaviours to target in the workplace with scant health promotion resources available. Additional strengths of the study design include cost-effectiveness of the approach, compared to longitudinal studies, and not being impacted by attrition over time (Pan, 2021). In additional, other strengths include the objective measure of weight, height, waist, and blood pressure at Time 1. Validated and previously published survey questions and tools were also utilised to examine health behaviours, sleep, mental health, and type 2 diabetes risk.

Several limitations are acknowledged. The study design was cross-sectional and represents a snapshot at two timepoints, so causality cannot be determined (Blake et al., 2011; Holton et al., 2021a, 2021b), nor were participants able to be followed longitudinally due to the burden of COVID-19 on staff (furloughing, illness, burnout, changing rosters) and ever-changing policy directives from the state government. The sample at both time points could have been subject to selection bias (Happell et al., 2014; Mittal et al., 2018; Pascoe et al., 2022) and may be considered small, particularly at Time 2. There is a lack of knowledge about non-responders; therefore, caution should be taken when generalising to the wider workforce (Blake et al., 2011; Holton et al., 2021a, 2021b). In order to capture current health behaviours during the stress of COVID-19, a shortened survey was conducted. Additionally, due to the spontaneous and unexpected nature of COVID-19 (Smallwood et al., 2021), including frequently-changing social distancing restrictions and secondment of clinical practitioners, the survey at Time 2 was collected wholly online. Therefore, objective data were self-reported, and blood pressure was not recorded, which impacted calculation of type 2 diabetes risk at Time 2.

The data collected may be at risk of self-report bias (Blake et al., 2011). There is also a risk of social desirability bias; therefore, reported behaviours could be more favourable than reality (Schneider et al., 2019). Self-report anthropometry is a limitation at Time 2 and may underestimate prevalence of overweight and obesity (Bogossian et al., 2012). However, this is a well-accepted method, and other survey questions were based on validated and widely published survey tools (Department of Health, 2020; Kessler et al., 2003; Victorian Agency for Health Information, 2021).

Sex distribution was largely female, (although representative of this health service's workforce), so findings may not represent those of male healthcare workers. This occupational gender distribution is typical, particularly for nursing and similar to other studies (Bogossian et al., 2012; Pascoe et al., 2022). Recruitment was via internal email within the health service and via advertisements; therefore, response rates are conservative and based upon number of staff who consented, then completed the survey and not the number who received a link to the survey (Holton et al., 2021b). This study was conducted at a large rural health service, and the sample was largely homogenous; therefore, results may not be generalisable to metropolitan health services (Holton et al., 2021b) nor staff who are born overseas. Time 2 data represent one point in time of the pandemic, and since data collection, there have been ongoing impacts of new COVID-19 strains, mandatory vaccination, and transition to living in “covid-normal” times (Pascoe et al., 2022). Future regular monitoring of the impacts of continued workplace disruptions (Pascoe et al., 2022) on healthcare staff is recommended.

## 6 Conclusion

There is clear recognition of the importance of supporting healthy behaviours and focussing on the health and wellbeing of healthcare staff. This is never more evident than in the current post-COVID-19 healthcare crisis. Similar to the general population, a high proportion of staff were living with overweight/obesity and had risk factors for future chronic disease. These risk factors included suboptimal compliance with fruit, vegetable, and physical activity guidelines and experiencing psychological distress. There are clear leverage points for intervention in the workplace to enable healthier behaviours and support positive mental health and wellbeing. Broadening the research into improving the health and wellbeing of healthcare workers in general should be a key thrust of future workforce studies. These improvements should be seen as fundamental to recruitment, retention, and attraction strategies into the future. This will optimise health and retainment of staff. Longitudinal research tracking the continued impact of COVID-19 in health and wellbeing of healthcare staff is advised.

## Funding sources

The data collection was funded in Time 1 by a 2018 Deakin University School of Health and Social Development School Research Grant. The remaining portion of the study was conducted in kind, with no funding to declare. The funder did not have a role in designing the study nor the data collection and interpretation of findings.

## Ethics approval and consent to participate

Approval to conduct this study was given by the Deakin University Human Ethics Advisory Group (HEAG-H 138_2018). Informed written consent was obtained from all participants in this study.

## Data availability

The datasets generated and analysed during the current study are not publicly available due to ethical constraints on data sharing (i.e., participants in this study did not consent to their data being shared with third parties).

## CRediT authorship contribution statement

**Kristy A Bolton:** Writing – review & editing, Writing – original draft, Supervision, Project administration, Methodology, Investigation, Funding acquisition, Formal analysis, Data curation, Conceptualization. **Penny Fraser:** Writing – review & editing, Supervision, Project administration, Methodology, Data curation, Conceptualization. **Steven Allender:** Writing – review & editing. **Rohan Fitzgerald:** Writing – review & editing, Supervision, Project administration, Methodology. **Susan Brumby:** Writing – review & editing, Project administration, Methodology.

## Declaration of competing interest

None.

## Appendix Supplementary materials

Image, application 1
