# Supplementary material for: How healthy are the healthcare staff in a rural health service? A cross-sectional study
Source: Int J Nurs Stud Adv. 2024 Feb 24;6:100186. doi: 10.1016/j.ijnsa.2024.100186 (PMC11080437; doi:10.1016/j.ijnsa.2024.100186)
Supplement: Supplementary file 1 [file mmc1.docx]

**Supplementary Material Table 1: Self-assessed health status of participating staff at Time 1**

|  | Time 1 2018 | |
| --- | --- | --- |
|  | *n* | % |
| **In general, how would you describe your health?** | | |
| Excellent | 18 | 9.78 |
| Very good | 56 | 30.43 |
| Good | 78 | 42.39 |
| Fair | 25 | 13.59 |
| Poor | 7 | 3.80 |
| **How much bodily pain have you had during the past 4 weeks?** | | |
| None | 32 | 17.39 |
| Very mild | 95 | 51.63 |
| Moderate | 51 | 27.72 |
| Severe | 6 | 3.26 |
| very severe | 0 | 0 |
| **How much did your health interfere with your normal activities (outside and/or inside the home) during the past 4 weeks?** | | |
| Not at all | 115 | 62.50 |
| Slightly | 50 | 27.17 |
| Moderately | 13 | 7.07 |
| Quite a bit | 6 | 3.26 |
| **How happy are you with your weight and size?** | | |
| Very unhappy | 20 | 10.87 |
| Unhappy | 39 | 21.20 |
| In between/okay | 73 | 39.67 |
| Happy | 43 | 23.37 |
| Very happy | 9 | 4.89 |
| **In the last 12 months have you tried to lose weight?** | | |
| Yes | 109 | 59.24 |
| **AUSDRISK – risk of developing type 2 diabetes within 5 years** | | |
|  | *n* | % |
| Low risk | 50 | 28 |
| Intermediate risk | 70 | 39 |
| High risk | 60 | 33 |

*n:* number of participants

**Supplementary Material Table 2*:* Level of psychological distress as measured by Kessler 10** **in participants who completed the survey**

|  | Time 1 2018 | | Time 2 2020 | | Time 1 vs Time 2 |
| --- | --- | --- | --- | --- | --- |
| **Kessler 10** **level of psychological distress (categories)** | | | | |  |
|  | *n* | % | *n* | % | *p* value* |
| Low ≤20 | 106 | 58 | 36 | 41 | 0.012* |
| Moderate≥20-24 | 48 | 26 | 26 | 30 |  |
| High≥25-≤29 | 26 | 14 | 17 | 20 |  |
| Very high≥30 | 4 | 2 | 8 | 9 |  |

*n:* number of participants; **p* value by chi-square or independent t-test where appropriate, whereby *p*<0.05 was considered statistically significant.

**Supplementary Material Table 3: COVID-19 specific data collected from participating staff at Time 2**

|  | Time 2 2020 | |
| --- | --- | --- |
| **Prior to COVID-19 did you work from home?** | *n* | % |
| Not at all | 71 | 93 |
| Some of the time | 5 | 7 |
| **Compared to pre- COVID-19 have you worked from home?** | | |
| More during Covid -19 | 14* | 100 |
| **What is your COVID-19 status?** | |  |
| No known direct contact with Covid -19 | 73* | 100 |
| **During COVID-19, do you think you have…** | | |
| Put on weight | 33 | 46 |
| Stayed about the same | 29 | 40 |
| Lost weight | 10 | 14 |
| **Compared to pre-COVID-19, has the food you have eaten during COVID-19 been…** | | |
| Much more than usual | 11 | 13 |
| About usual | 66 | 76 |
| Much less than usual | 10 | 12 |
| **Compared to before COVID-19, has the amount of physical activity you have done during COVID-19 been…** | | |
| Much more than usual | 11 | 15 |
| About usual | 46 | 61 |
| Much less than usual | 19 | 25 |
| **During COVID-19, have you participated in any of the Western District Health Service COVID-19 health and wellbeing activities?** | | |
| Yes | 29 | 41 |

*n:* number of participants; Note 100% reflects the number of participants who responded to the question.

**Supplementary Material Table 4: Perceived impact of COVID-19 on participating staff at Time 2**

| **What is the impact of COVID-19 on different areas/aspects of life:** | | | | | | | | | | | | | | |
| --- | --- | --- | --- | --- | --- | --- | --- | --- | --- | --- | --- | --- | --- | --- |
|  | Negative impact | | | | | No impact | | | | Positive impact | | | | |
|  | *n* | | % | | | *n* | | % | | *n* | | | % | |
| Social connection | 47 | | 67 | | | 20 | | 29 | | 3 | | | 4 | |
| Stress | 55 | | 78 | | | 11 | | 16 | | 5 | | | 7 | |
| Studies | 18 | | 31 | | | 37 | | 63 | | 4 | | | 7 | |
| Family relationships | 34 | | 49 | | | 30 | | 43 | | 6 | | | 9 | |
| Friend relationships | 39 | | 55 | | | 27 | | 38 | | 5 | | | 7 | |
| Employment | 22 | | 32 | | | 43 | | 62 | | 4 | | | 6 | |
| Finances | 14 | | 19 | | | 42 | | 58 | | 16 | | | 22 | |
| Exercise | 25 | | 35 | | | 33 | | 47 | | 13 | | | 18 | |
| Sleep | 34 | | 47 | | | 35 | | 48 | | 4 | | | 6 | |
| Diet | 26 | | 37 | | | 39 | | 55 | | 6 | | | 9 | |
| **With regards to COVID-19 currently how stressed are you about each of the following?** | | | | | | | | | | | | | | |
|  | Not at all stressed | | | Slightly stressed | | | Moderately stressed | | Very stressed | | | Extremely stressed | | |
|  | *n* | % | | *n* | % | | *n* | % | *n* | | % | *n* | | % |
| **Childcare** | 26 | 67 | | 6 | 15 | | 4 | 10 | 0 | | 0 | 3 | | 8 |
| **Home schooling** | 23 | 50 | | 10 | 22 | | 6 | 13 | 3 | | 7 | 4 | | 9 |
| **Elderly** | 21 | 40 | | 12 | 23 | | 13 | 25 | 6 | | 11 | 1 | | 2 |
| **Access to food** | 50 | 76 | | 11 | 17 | | 4 | 6 | 1 | | 2 | 0 | | 0 |
| **Infection** | 18 | 27 | | 28 | 42 | | 14 | 21 | 3 | | 5 | 3 | | 5 |
| **Family infection** | 10 | 15 | | 32 | 46 | | 14 | 20 | 7 | | 10 | 6 | | 9 |
| **Keeping your job** | 44 | 64 | | 15 | 22 | | 7 | 10 | 2 | | 3 | 1 | | 2 |
| **Finances** | 36 | 51 | | 24 | 34 | | 4 | 6 | 3 | | 4 | 4 | | 6 |

*n:* number of participants
